# In Situ Ambient Pressure Photoelectron Spectroscopy Study of the Plasma–Surface Interaction on Metal Foils

**DOI:** 10.1021/acs.langmuir.4c01102

**Published:** 2024-06-25

**Authors:** Sam Taylor, Filip Hallböök, Robert H. Temperton, Jinguo Sun, Lisa Rämisch, Sabrina Maria Gericke, Andreas Ehn, Johan Zetterberg, Sara Blomberg

**Affiliations:** † Division of Chemical Engineering, Lund University, 223 62 Lund, Sweden; ‡ MAX IV Laboratory, Lund University, 224 84 Lund, Sweden; § Division of Combustion Physics, Lund University, 221 00 Lund, Sweden

## Abstract

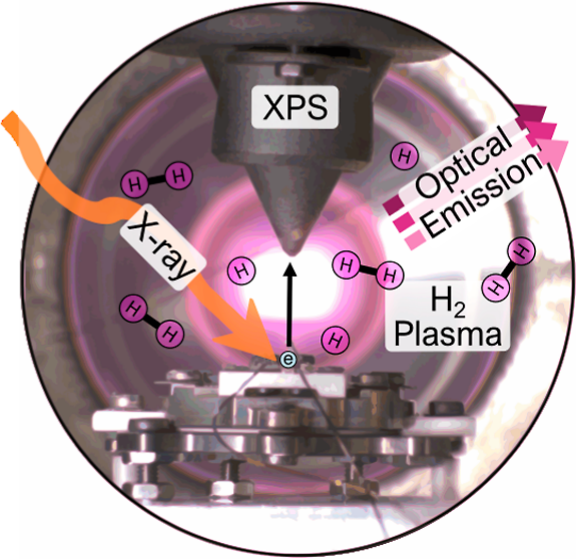

The plasma–surface interface has sparked interest due to its potential of creating alternative reaction pathways not available in typical gas–surface reactions. Currently, there are a limited number of in situ studies investigating the plasma–surface interface, restricting the development of its application. Here, we report the use of in situ ambient pressure X-ray photoelectron spectroscopy in tandem with an optical spectrometer to characterize the hydrogen plasma’s interaction with metal surfaces. Our results demonstrate the possibility to monitor changes on the metal foil surface in situ in a plasma environment. We observed an intermediate state from the metal oxide to an –OH species during the plasma environment, indicative of reactive hydrogen radicals at room temperature. Furthermore, the formation of metal-carbides in the hydrogen plasma environment was detected, a characteristic absent in gas and vacuum environments. These findings illustrate the significance of performing in situ investigations of the plasma–surface interface to better understand and utilize its ability to create reactive environments at low temperature.

## Introduction

Low-temperature plasmas are increasingly becoming more prevalent in various research and industrial applications for material and chemical modification. For example, hydrogen plasma for mineral and metal ore reduction processes is being investigated as it potentially offers advantages over traditional hydrogen-gas processes due to the high reactivity of the plasma helping overcome the reaction barriers.^1−6^ These nonthermal plasmas are able to overcome these reaction barriers by accessing high-temperature chemistry without excessive gas heating. However, despite the significant number of empirical and theoretical studies examining the plasma–surface phenomena, little progress has been made in probing the plasma–surface interaction due to its high underlying complexity.^7^ A better understanding of the plasma–surface interaction would allow further optimization of the plasma and surface conditions to further improve the reaction kinetics.^7^

Investigations into this plasma–surface interface are therefore greatly sought to deepen our understanding of the surface chemistry occurring in a plasma environment. Although in situ techniques such as Raman spectroscopy^8,9^ and IR spectroscopy^10^ have been applied, one powerful chemical analysis technique for surface characterization currently not implemented in situ is X-ray photoelectron spectroscopy (XPS), as it is traditionally operated in a high vacuum. As such, previous investigations on the plasma–surface interface perform ex situ measurements by exposing the sample to the plasma environment before returning to vacuum conditions for XPS measurements.^11−13^ While these ex situ XPS measurements provide vital information regarding the post-plasma surface chemistry, it cannot fully describe the intermediate chemical reactions or temporary chemical states that occur during the plasma environment. Ambient pressure XPS, however, enables measurements in gas environments with pressures typically in the order of mbars. Crucially, these pressures have allowed the possibility of performing AP-XPS in nonthermal plasma conditions.^14^

This study aims to further investigate the viability of performing in situ AP-XPS measurements in a plasma environment. Specifically, we will focus on the interaction of radio frequency generated hydrogen plasma on two transition metals. The transition metals, nickel (Ni) and cobalt (Co), are used due to their relevance in plasma–surface related applications.^3,7,15−17^ These results will showcase the ability to extract further information behind the plasma–metal interaction, which is key to further develop plasma-based applications, e.g., plasma catalysis.^7,18^

## Experimental Section

The AP-XPS measurements were completed at the HIPPIE beamline at the MAX IV Synchrotron Laboratory, Lund, Sweden. MAX IV’s HIPPIE provides two complementary endstations, and both were utilized during this experiment. The solid–gas endstation was used for the sample preparation, while the solid–liquid endstation was utilized for the AP-XPS measurements under hydrogen plasma conditions.

The effect of hydrogen plasma on metal surfaces was investigated by using nickel (Ni) and cobalt (Co) foils. Each metal surface was consecutively studied in four environmental conditions: (i) vacuum, (ii) hydrogen gas, (iii) hydrogen plasma, and (iv) vacuum. The sample was initially placed into a vacuum environment of pressure 10^–5^ mbar, followed by flowing hydrogen gas into the chamber to a pressure of 10^–1^ mbar. After that, the hydrogen gas was ignited to form a plasma by using a 13.56 MHz radio frequency generator (RF-generator) at 300 W (SVT associates). Finally, the environment was returned back to vacuum conditions such that the initial and final states of the sample could be compared. These four environmental conditions provide the foundation to understand how the plasma influences the metal surface.

In each of the four conditions, the O 1s and the metal 2p_3/2_ regions were recorded using a photon energy of 1500 eV, and the C 1s region was recorded using a photon energy of 600 eV. The hydrogen radicals generated in the plasma are considered to have a penetration depth larger than all of the probing depths of the incoming photons.^19,20^

### Sample Preparation

The nickel and cobalt metal foil (99.9% purity from Sigma-Aldrich) were cut to 5 × 5 mm samples and cleaned in an ultrasonic bath of acetone for 10 min before being rinsed with ethanol. These samples were then placed in HIPPIE’s solid–gas endstation for preparation. The samples were initially sputtered with argon, removing surface contamination, before heated to 600 °C in vacuum.

**Figure 1:**
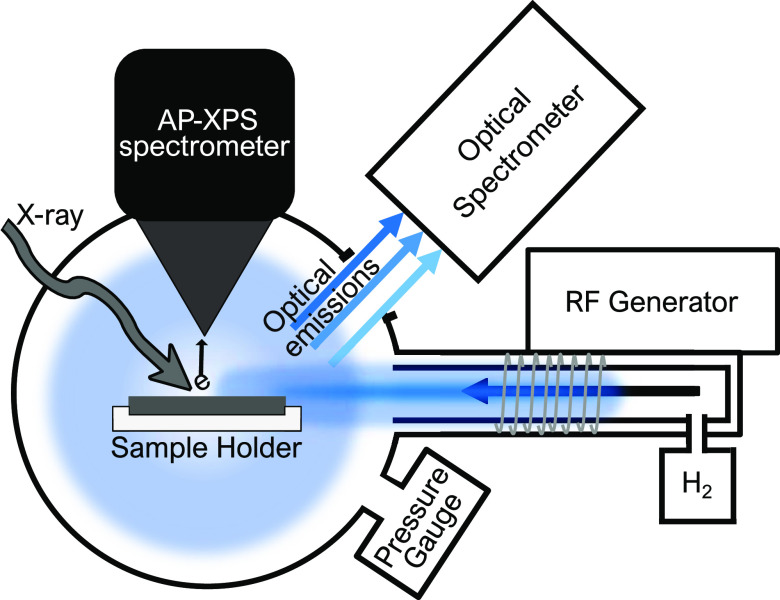
HIPPIE’s solid–liquid endstation setup adapted with an RF generator and optical spectrometer for plasma experiments (viewed from above).

Afterward, the sample was transported through the air to HIPPIE’s solid–liquid endstation to perform AP-XPS. This endstation, equipped with a SPECS Phoibos 150 NAP hemispherical electron analyzer, was used due to its large backfilled chamber with appropriate space to mount the plasma source and optical spectrometer. The sample, now in the chamber at the solid–liquid endstation, is attached to a grounded sample holder and spot-welded to the thermocouple. The sample is then positioned at the normal emission angle with the surface pointing straight toward the electron analyzer and held at room temperature for the measurements. The final setup can be seen in the schematics in Figure 1 below.

### Surface and Environment Characterization

#### Surface Characterization

The emitted core electrons were detected by an AP-XPS spectrometer, and the nozzle of which is shown in Figure 1. Each XPS spectra is calibrated to the Fermi edge for the corresponding excitation energy and normalized to the spectra’s maximum. After which, the spectra were fitted to a Shirley background and subsequently subtracted from the data before the SkewedVoigt peaks were fitted using the lmfit python package.^21^ Note that, however, only the metallic peaks were fitted with an asymmetric line shape. Given the different environmental conditions, the fitting procedure allowed the peak’s fwhm to vary by a maximum of ±0.18 eV and the peak position by a maximum of ±0.2 eV, with the exception of a few notable peaks mentioned later. The measurement process took around 5 min per environment to collect all the necessary data, and as such, each metal sample was exposed to approximately 5 min of hydrogen plasma. The effect of the beam on the sample was considered by performing multiple scans for a single measurement. No discrepancies between the scans were noticed.

#### Environment Characterization

The grade 5 hydrogen gas flowed into the RF-generator operating at 13.56 MHz, and the chamber’s pressure was controlled via a leak valve until 10^–1^ mbar was reached. The RF-generator, set to 300 W of input power, was ignited when needed to create a hydrogen plasma environment. To remove the plasma, the input power was switched off.

The hydrogen plasma environment was characterized using an optical spectrometer (Princeton Instruments, IsoPlane-160) measuring between 300 and 750 nm with a grating of 150 lp/mm. The resultant optical emission spectra were recorded via an attached camera (Andor iStar ICCD). The optical spectrometer and corresponding camera were installed on a quartz viewport of the experimental chamber pointing toward the sample surface. A background spectrum was obtained and subtracted from the plasma optical emission spectra prior to plasma ignition.

## Results and Discussion

### AP-XPS in Plasma

Survey spectra of the two samples were measured in hydrogen plasma to verify that AP-XPS measurements can be carried out in situ with the plasma turned on.

**Figure 2:**
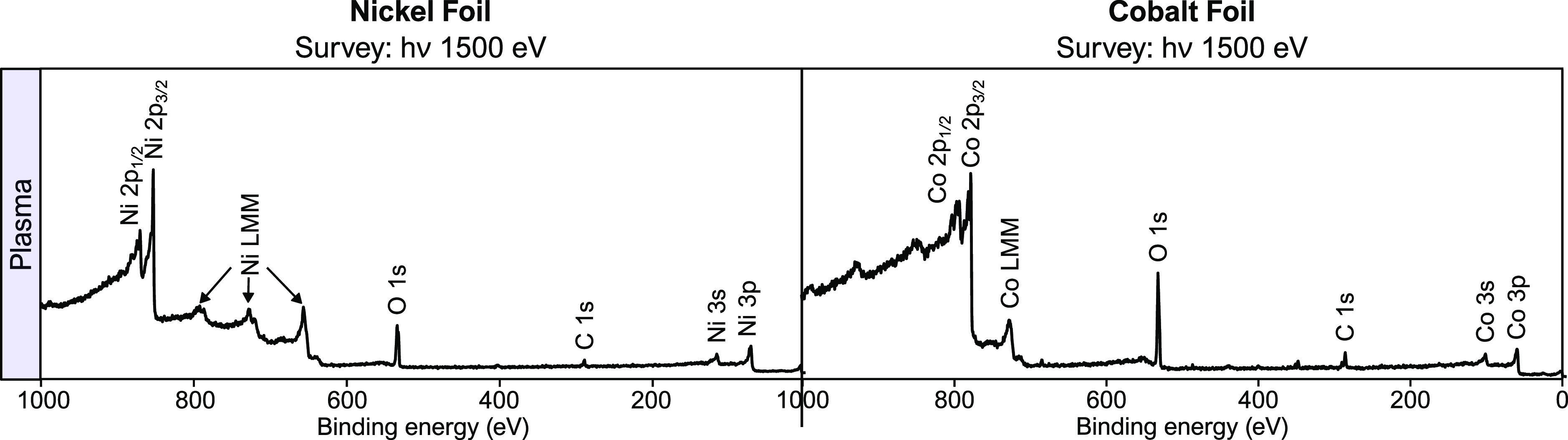
Labeled survey spectra of nickel and cobalt foil in the hydrogen plasma environment. All major peaks are assigned in the figure.

Figure 2 shows the nickel and cobalt foil XPS survey spectra. The metal peaks Ni 2p_3/2_ (853 eV) and Ni 2p_1/2_ (868 eV) as well as Co 2p_3/2_ (778 eV) and Co 2p_1/2_ (793 eV) are labeled accordingly. Additionally, the O 1s (528 eV) and the C 1s (286 eV) are also labeled.

The fact that the assigned peaks’ binding energy are consistent with previously reported binding energies^22^ implies that the RF plasma does not have a drastic influence on peak position. Similarly, there are no other obvious artifacts from the plasma on the emitted electrons, even at low kinetic energy, suggesting that there is minimal interaction between the plasma and the detected electrons. Although, changes in the line shape or subtle peak shifts are not visible in the survey spectra. A follow up test demonstrated that there is no peak shift inherent to the RF-generated plasma environment, as shown in Figure S1 of the Supporting Information.

**Figure 3:**
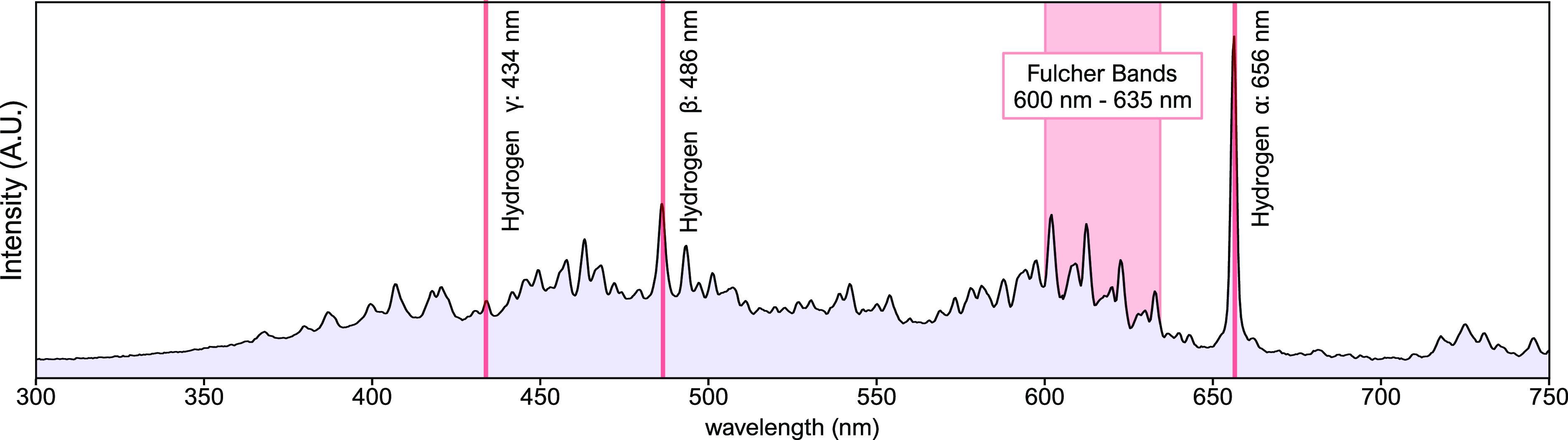
Optical emission spectra of the RF-generated hydrogen plasma at 300 W.

Additionally, the plasma properties were characterized via the optical emission spectra shown in Figure 3.

From the optical emission spectra (Figure 3), hydrogen plasma is evidently present in the chamber. The presence of the alpha, beta, and gamma transition lines show that the hydrogen molecule has been separated into electronically active hydrogen atoms. Additionally, the presence of the hydrogen molecule’s roto-vibration Fulcher bands shows that the hydrogen also exists as vibrationally excited molecules in the plasma.^6,23^ These signals demonstrate a very diverse and active hydrogen plasma environment.

### Plasma–Surface Interaction

#### Nickel Surface

**Figure 4:**
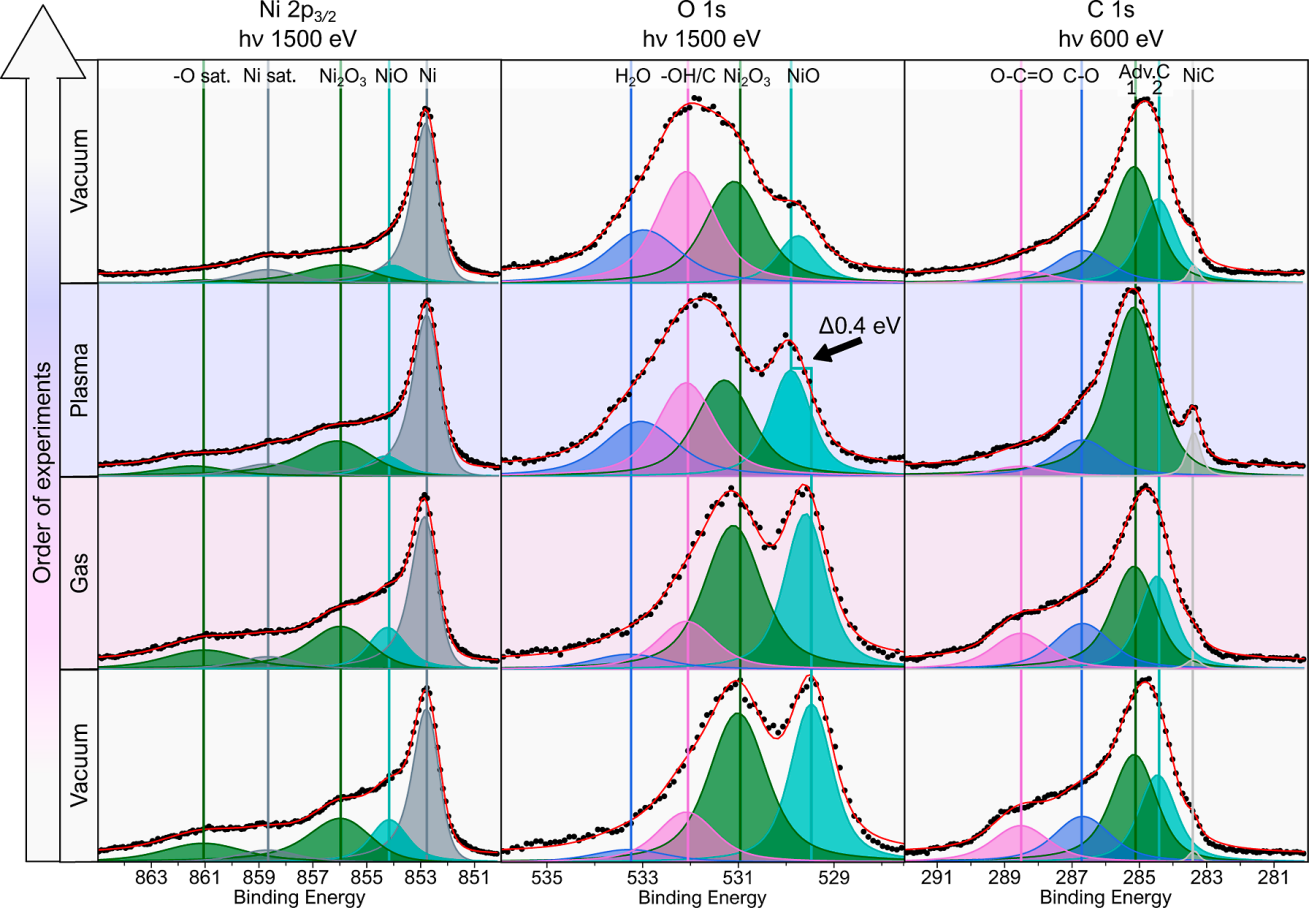
Measured room temperature nickel foil XPS regions (Ni 2p_3/2_, O 1s, and C 1s) under different environments. The black dots show the experimental data after the subtraction of the Shirley background, and the red line is the resulting line shape from the peak fitting.

Figure 4 shows the Ni 2p_3/2_, O 1s, and C 1s spectra obtained from the nickel foil in the initial vacuum, hydrogen gas, hydrogen plasma, and final vacuum environment (bottom to top).

Focusing on the Ni 2p_3/2_ region (Figure 4) in the initial vacuum environment shows several photoemission peaks and corresponding satellites. The most intense peak is assigned to the metal bulk at 852.6 eV.^24^ Given the prominence of the nickel bulk peak, the formed oxide layer is noted to be thin. The shoulder of the metal bulk peak is decomposed into two oxide peaks of NiO at 854.1 eV and Ni_2_O_3_ at 855.6 eV.^25−29^ While there is some discussion of the origin behind the Ni_2_O_3_ peak, either due to NiO satellite, Ni vacancies in the NiO surface lattice, a nickel hydroxide [e.g., NiOOH or Ni(OH)_2_] or sputter-induced defects,^28−33^ for the purpose of this study, we will refer to this peak as Ni_2_O_3_. Note that this is a broad peak that encapsulates many different aspects of the nickel surface. Additionally, two separate satellite peaks are identified in the spectra: one at 858.6 eV from the metal bulk and another at 861.2 eV from the oxides.

As expected, exposing the nickel foil to hydrogen gas at room temperature does not change the ratio of the previously mentioned peaks. However, when the plasma is ignited, there is a decrease in the NiO peak and a very slight decrease in the Ni_2_O_3_ peak. There could also be hydroxides forming on the surface, which would appear as a peak in the same binding energy region. After plasma ignition, in the final vacuum state, the Ni_2_O_3_ peak has also reduced relative to the metal bulk peak. Additionally, there is a visible Ni satellite peak that is no longer saturated by the surrounding oxide or oxide satellite peaks. These trends indicate the oxide reduction of the metal foil.

To further understand the interaction between the hydrogen plasma and the Ni surface, one must examine the O 1s spectra (Figure 4). The O 1s region in the initial vacuum environment shows two distinct oxide peaks relating to the lattice NiO at 529.5 eV and the surface Ni_2_O_3_ at 531.0 eV, similar to the Ni 2p_3/2_ region. The spectrum is also fitted with the component assigned to hydroxides and carboxides, represented by the –OH/C peak at 532.1 eV.^33−36^ During the hydrogen plasma environment, the spectrum has an overall shift toward higher binding energies, which is interpreted as the partial reduction of lattice oxides NiO toward surface hydroxides (−OH) and water, shown by the H_2_O peak at 533.3 eV.^35^

The observed shift of the NiO peak has previously been reported as NiO_1–*x*_–OH at 530.0 eV, relating to an OH molecule adsorbed on an oxide vacancy (hence the subscript 1 – *x*).^33^ As such, we interpreted this peak as a nickel oxide reacting with a hydrogen radical. Therefore, the oxygen in the nickel oxide, when interacting with hydrogen radicals, is reduced to a loosely bound OH species, potentially becoming either surface hydroxides or H_2_O, which can then desorb. A similar shift toward higher binding energy occurs for the Ni_2_O_3_ peak. However, given this peak’s close proximity to the –OH/C peak, it is difficult to resolve the individual contributions of the OH/C peak and Ni_2_O_3_ peak on the spectra.

Finally, one can examine hydrogen’s interaction with carbon contaminants on the surface. The C 1s region (Figure 4) is initially dominated by adventitious carbon (Adv.C), labeled as two peaks Adv.C 1 and Adv.C 2 at 284.5 and 285.1 eV, respectively.^37−39^ The adventitious carbon is marked by two peaks due to the notable lack of Adv.C 1 at high temperature,^40^ as shown in Figure S1 in the Supporting Information, as well as to account for spectrum change during the plasma environment, as shown in Figure 4. The C–O and O–C=O peaks are assigned at 286.6 and 289 eV, respectively. The O–C=O peak has a counterpart peak in the O 1s regions between 531.5 and 532 eV, including the –OH/C peak. This O–C=O peak disappears entirely after being exposed to hydrogen plasma. The other prominent peak during the hydrogen plasma is assigned to nickel carbide (NiC) at 283.4 eV.^29,41,42^ This NiC peak is not assigned in the Ni 2p_3/2_ region due to the relatively low intensity of the C 1s region (see Figure 2). There is an increase in the Adv.C 1 peak and the NiC peak during the hydrogen plasma environment, showing that the radicals are interacting with the loosely bound carbon molecules,^43^ potentially allowing for other bonds to occur such as the NiC peak. However, it is difficult to determine whether NiC was always present but hidden by the Adv.C 2 peak. After exposure to the plasma, the relative intensity of the NiC and Adv.C 1 peaks is reduced, and the Adv.C 2 peak returns, suggesting that the hydrogen plasma interaction with the carbon bonds is temporary and requires in situ methods to be detected. When the sample is back in vacuum conditions, a signal count increase of the carbon peak was observed, which we correlate to carbon species that has desorbed from the walls of the chamber under plasma conditions, now deposited on the sample.

#### Cobalt Surface

A second experiment was performed on cobalt foil to examine if the material’s properties have an impact on hydrogen plasma–surface interaction. The cobalt foil was exposed to the same experimental procedure as that for the nickel foil.

**Figure 5:**
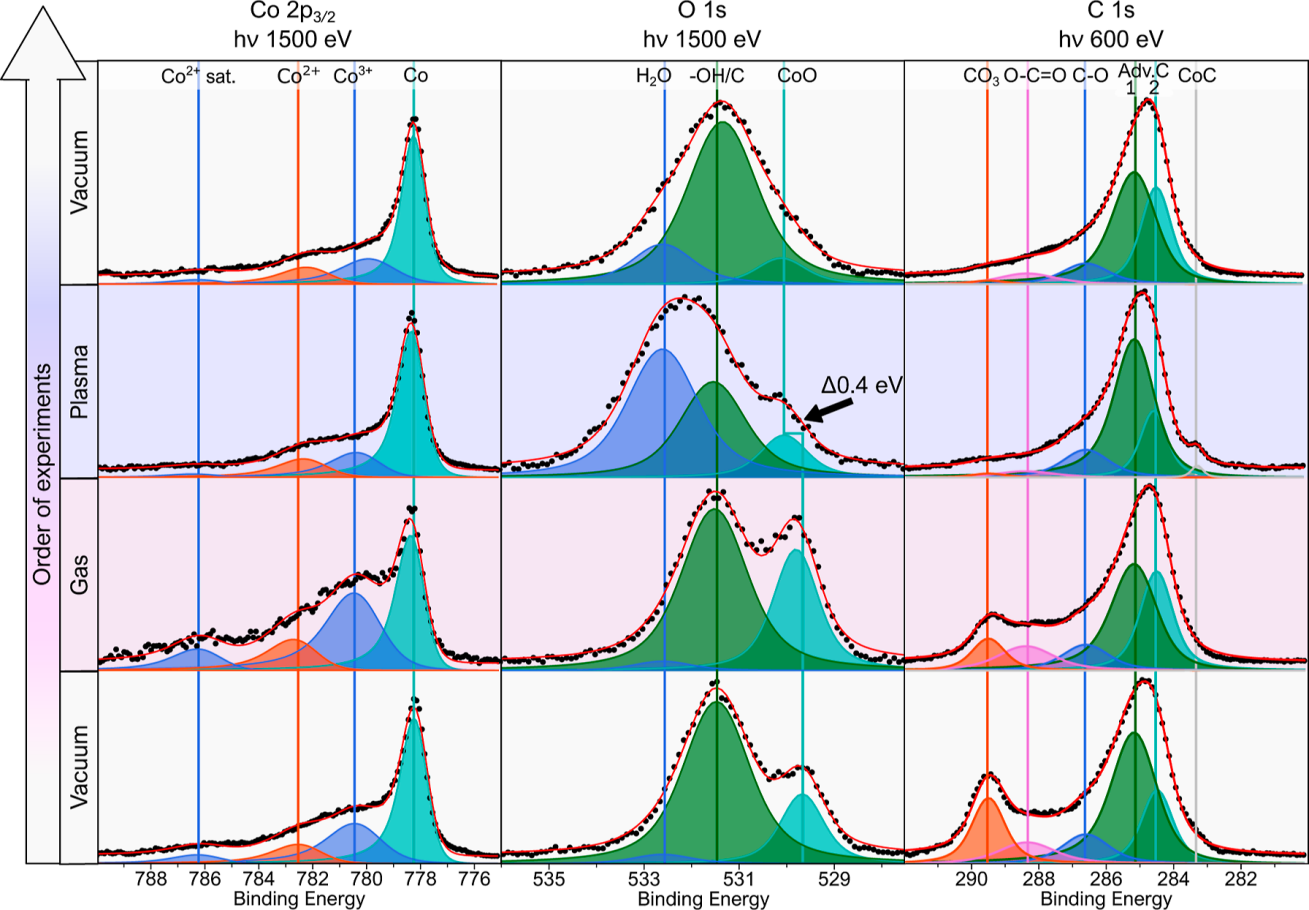
Measured room temperature cobalt foil XPS regions (Co 2p_3/2_, O 1s, and C 1s) under different environments. The black dots show the experimental data after the subtraction of the Shirley background, and the red line is the resulting line shape from the peak fitting.

Figure 5 shows the Co 2p_3/2_ spectra, O 1s spectra, and C 1s spectra in vacuum, gas, and plasma environment.

The most intense peak in the Co 2p_3/2_ region (Figure 5) is the bulk Co 2p_3/2_ peak at 778.1 eV, which remains detected throughout all environments.^42,44,45^ Due to the challenge of assigning peaks in this region to specific oxide structures, the peaks are instead assigned to Co^2+^ and Co^3+^ at 780.2 and 782.4 eV, respectively.^46^

Surprisingly, the Co^2+^ and Co^3+^ peaks increase in intensity relative to the bulk peak when flowing the hydrogen gas. This is difficult to explain, but it may potentially be due to water contamination in the hydrogen gas or water contamination in the chamber. However, these contaminants are quickly removed during plasma exposure, demonstrating the reactivity and, thereby, the cleaning ability of hydrogen plasma on cobalt foil. Therefore, analogous to the nickel oxide reduction, the cobalt oxide has reduced as a result of the hydrogen plasma exposure at room temperature.

The plasma’s interaction with oxygen-related surface species can be further studied in the O 1s region (Figure 5). The initial vacuum phase contains two prominent peaks, one being the peak CoO, assigned at 529.6 eV,^47^ the other peak at 531.5 eV is more challenging to assign. The peak at 531.5 eV, labeled –OH/C, contains contributions from the −OH_ad_ as well as the C–O peaks. However, the Co_2_O_3_ component also appears at a similar binding energy.^35^ During exposure to hydrogen gas, there is an increase in the CoO peak intensity, similar to that of the Co 2p_3/2_ region, reaffirming the contamination caused by flowing hydrogen gas. However, both the CoO and the –OH/C peak decrease in intensity during exposure to hydrogen plasma, with an additional peak at 532.6 eV appearing, attributed to the formation of H_2_O on the surface.^45,48^ Interestingly, a binding energy shift similar to that of the NiO → NiO_1–*x*_–OH (≈0.4 eV) is seen in the CoO peak. As such, this peak shift is interpreted as cobalt oxide, which reacts with a hydrogen radical (denoted as CoO_1–*x*_–OH). Finally, after plasma exposure, the environment is returned to vacuum conditions. The resulting H_2_O peak has substantially decreased, as well as a reduction in the oxide peaks CoO/CoO_1–*x*_–OH such that only the −OH_ad_ peak remains as the main feature of the spectra. Despite the difficulties in interpretation, the majority of the –OH/C peak is most likely due to a combination of the −OH_ad_ and the Co_2_O_3_ state and not C–O on the surface due to the hydrogen cleaning of C–O. However, as previously mentioned, it is difficult to isolate the effect from water contamination from the chamber and from the hydrogen gas. One can further demonstrate this by examining the C 1s region (Figure 5) under different conditions. Similar to nickel foil’s C 1s region, we initially have adventitious carbon on the surface as signified by the peaks Adv.C 1 and Adv.C 2 at 284.5 and 285.1 eV, respectively. The four other peaks of CO_3_, O–C=O, C–O, and CoC are assigned to 289.5 288.4, 286.6, and 283.3 eV, respectively.^42,47,49−52^ The Adv.C 1 peak as well as the CO_3_ peak present during the initial vacuum condition seemingly decreases in the presence of hydrogen gas. During the plasma phase, peaks assigned to carbon binding with oxygen at the surface are almost completely removed, and the Adv.C 1 and carbide both increases, illustrating the strong interaction between the hydrogen plasma and the carbon species. In the final vacuum conditions, the main features of the spectra are the Adv.C 1 and Adv.C 2 peaks, which are noted to come from carbon contamination from the chamber walls.

### Plasma–Surface Reactions

In this study, the plasma–surface has been probed using AP-XPS, demonstrated by in situ monitoring of hydrogen plasma interaction with Ni and Co metals. While differences between the metal foils are present, it is difficult to assess if these differences arise from fundamental aspects of the metal’s characteristics or from differences in the environmental conditions, leading to changes in the XPS spectra. Further experiments focused on investigating these differences will be important when assessing a metal interaction with plasma. However, the Ni/Co 2p_3/2_ and O 1s spectra of both metals demonstrated the oxide reduction ability of the hydrogen plasma. These findings are in agreement with previous ex situ XPS studies examining hydrogen plasma interaction with transition metals.^11,12^ Notably, the exposure to hydrogen plasma made it possible to reduce the metal oxides at room temperature due to the plasma’s high reactivity. As a result of this reduction, H_2_O is formed and thus observed in the O 1s spectrum, which aligns with reported post-plasma XPS measurements. However, we observed a significant difference in the recorded AP-XPS spectra while the plasma was in the chamber compared to post-plasma exposure, emphasizing the need for in situ analysis. The defect hydroxides (Ni/CoO_1–*x*_–OH), for example, are prominent during plasma conditions and suggest a fundamental mechanism in which the plasma is interacting with the surface. Interestingly, by studying the plasma–surface interaction, we can detect the lattice defect hydroxide during the hydrogen plasma environment which may be an active site for H_2_O production during reduction. Further investigations are still needed to determine how these defect hydroxides form and their importance in the reduction process. We propose that these lattice hydroxides are formed due to hydrogen radical reaction with the bulk Ni/CoO, forming Ni/CoO_1–*x*_–OH. Additional tests could also include taking measurements over an extended period of time to understand the temporal aspect of the plasma–surface interaction and these intermediate species. The metal carbide peak becomes a prominent feature in the spectra during the plasma environment, indicating a strong surface interaction with the hydrogen plasma and reaffirming the need for in situ measurements of the surface during plasma conditions.

## Conclusions

AP-XPS has been successfully used in situ to examine the plasma–surface interface while simultaneously performing optical emission spectroscopy to characterize the plasma. From our results, there seems to be little to no adverse effects inherent to using plasma with AP-XPS. As such, the in situ AP-XPS data obtained during the hydrogen plasma was used to demonstrate the reactivity of the hydrogen radicals on a room temperature sample, as well as detecting intermediate species due to the in situ configuration. Our measurements indicate a similar interaction between the hydrogen plasma and both metals, but more studies should be performed to thoroughly examine how the plasma influences the chemical reactions on the metal surface. The use of in situ AP-XPS in a plasma environment will play an invaluable part in deciphering the chemistry and intermediate species present in the plasma–surface interface, which will greatly increase our understanding of plasma-based applications such as plasma cleaning and plasma catalysis.^53^

## Supporting Information Available

Additional experiment regarding heated cobalt foil sample examining plasma charging effects (PDF)
