# Supplementary material for: In Situ Ambient Pressure Photoelectron Spectroscopy Study of the Plasma–Surface Interaction on Metal Foils
Source: Langmuir. 2024 Jun 25;40(27):13950–6. doi: 10.1021/acs.langmuir.4c01102 (PMC11238582; doi:10.1021/acs.langmuir.4c01102)
Supplement: Supplementary file 1 — la4c01102_si_001.pdf [file la4c01102_si_001.pdf]

In-situ ambient pressure photoelectron spectroscopy study of the plasma-surface interaction on metal foils

Sam Taylor,*,† Filip Hallböök,† Robert H. Temperton,‡ Jinguo Sun,¶ Lisa Rämisch,¶ Sabrina Maria Gericke,¶ Andreas Ehn,¶ Johan Zetterberg,¶ and Sara Blomberg*,†

†*Division of Chemical Engineering, Lund University, 223 62 Lund, Sweden*

‡*MAX IV Laboratory, Lund University, 224 84 Lund, Sweden*

¶*Division of Combustion Physics, Lund University, 221 00 Lund, Sweden*

E-mail: Sam.Taylor@ple.lth.se; Sara.Blomberg@ple.lth.se

Supplementary Information

Plasma charging effects

It is important to mention that the discussed shifts in peak positions are described as chemical shifts rather than charging effects due to plasma. This is justified by recognising that there are no shifts in the bulk nickel or bulk cobalt peak during plasma conditions. However, as the foils are grounded, one may expect that no significant shifts to occur, but not extend that reasoning to surface molecules or molecules insulated from the metal bulk. Many of the peak shifts described in the previous sections have been attributed to their reaction with a hydrogen radical in a plasma, which causes an increase in binding energy. Charging effects such as the removal of electrons on insulated materials also cause an increase in binding energy,[1] making it difficult to distinguish between potential plasma-based charging effects, and hydrogen chemistry.

As such, a final test on the cobalt foil was performed at an increased temperature. This foil was sputtered and annealed in the solid-gas endstation, similar to previous experiments. Once moved into the solid-liquid endstation, the temperature of the sample was increased and maintained to around 400 °C to remove and/or prevent significant amounts of oxide build up and desorb some of the surface contaminants.[2] By removing the metallic oxides and most surface contaminants, a more precise examination of the plasma charge effect can be studied on any remaining molecules. One such difficult to remove surface molecule is the adventitious carbon, which has often been used to correct for charge shifts in insulating materials. After which, the previous experimental procedure was followed.

Most notably for our discussion, the 284.5 eV Adv.C 2 peak in the C 1s region (Figure S1) doesn't appear to shift during exposure to the hydrogen plasma. This suggests that the increased temperature likely caused the desorption of loosely bound carbon, leaving behind stable carbon structures that remains unaffected by the hydrogen radicals.[3] This demonstrates that the plasma does not have an inherent charging effect on the surface

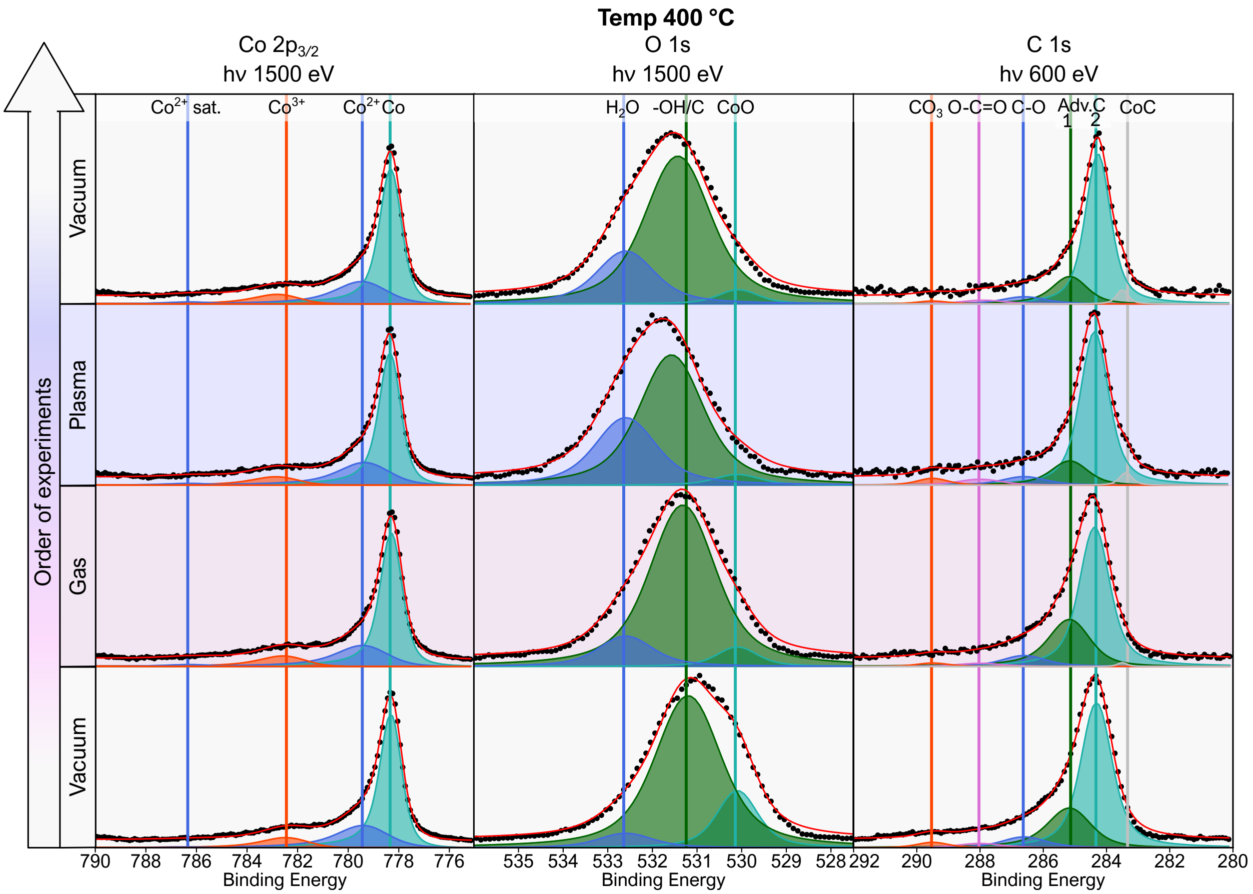

Figure S1: Measured cobalt foil at higher temperatures under different environmental conditions

molecules. Therefore, the previously described shift in peak positions can be attributed to chemical interaction with the plasma, and not a physical property of plasma, indicating the reliability of using the XPS technique in a plasma environment.

References

(1) Diulus, J. T.; Naclerio, A. E.; Boscoboinik, A.; Head, A. R.; Strelcov, E.; Kidambi, P. R.; Kolmakov, A. Operando Plasma-XPS for Process Monitoring: Hydrogenation of Copper Oxide Confined Under h-BN Case Study. *The Journal of Physical Chemistry C* **2024**,

(2) Antón, R. L.; González, J. A.; Andrés, J. P.; Canales-Vázquez, J.; Toro, J. A. D.; Riveiro, J. M. High-vacuum annealing reduction of Co/CoO nanoparticles. *Nanotechnology* **2014**, *25*, 105702.

(3) Park, S.; Siahrostami, S.; Park, J.; Mostaghimi, A. H. B.; Kim, T. R.; Vallez, L.; Gill, T. M.; Park, W.; Goodson, K. E.; Sinclair, R.; Zheng, X. Effect of Adventitious Carbon on Pit Formation of Monolayer MoS2. *Advanced Materials* **2020**, *32*, 2003020.
